# Chimeric antigen receptor T cell therapy for multiple myeloma

**DOI:** 10.1186/s41232-019-0100-6

**Published:** 2019-06-04

**Authors:** Kana Hasegawa, Naoki Hosen

**Affiliations:** Department of Cancer Stem Cell Biology, Osaka University Graduate School of Medicine, 1-7 Yamada-Oka, Suita, Osaka 565-0871 Japan

**Keywords:** CAR T cell, Immunotherapy, Multiple myeloma, Integrin

## Abstract

Chimeric antigen receptor (CAR) T cell therapy is a new cancer immunotherapy targeting cancer-specific cell surface antigen. CD19-CAR T cells have been already shown to be very effective to B cell leukemia/lymphoma. Now, many researchers are developing CAR T cells for multiple myeloma. CAR T cells targeting B cell maturation antigen (BCMA) showed promising efficacy in early phase clinical trials. We have recently reported that CAR T cells targeting the activated integrin β7 can selectively eradicate MM cells including CD19^+^ clonotypic B cells and are preparing a clinical trial.

## CAR T cell therapy

**Fig. 1:**
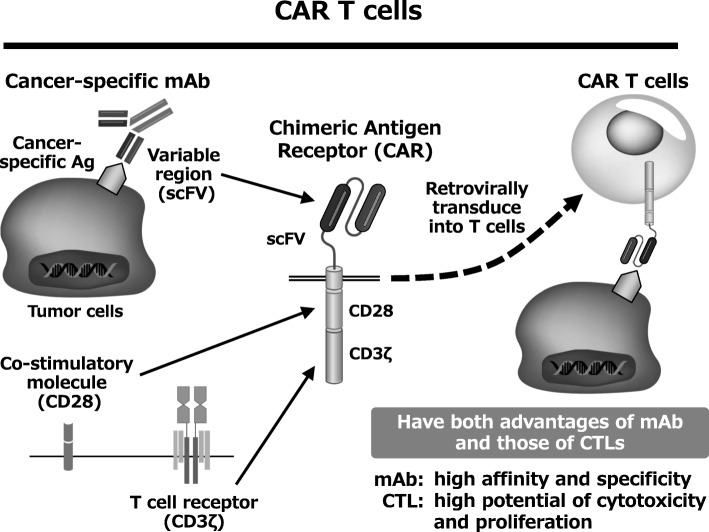
CAR T cells have both advantages of mAb and those of CTLs

CAR T cell therapy is a new cancer immunotherapy targeting cell surface antigens expressed on tumor cells. CAR is generated by fusing the antigen recognition domain of a tumor-specific monoclonal antibody (mAb) with CD3z and a co-stimulatory molecule (such as CD28 or 4-1BB). CAR T cells, which are established by transduction of CAR into T cells, are activated by recognizing the cancer cell surface antigen and kill cancer cells. CAR T cells have both advantages of mAb and those of cytotoxic T cells. CAR T cells have high affinity and specificity to tumor cells and also high potential of cytotoxicity and proliferation (Fig. 1).

In clinical trials of CD19 CAR T cells against acute lymphocytic leukemia and malignant lymphoma, very high complete remission rates were reported [1–3]. Consequently, CD19 CAR T cell therapy has been approved by the FDA in the USA in 2017. Severe adverse events such as cytokine release syndrome (CRS) and neurotoxicity are big problems. However, it has been shown that anti-IL6 receptor mAb is highly effective to CRS, and CAR T cell therapy is becoming safer. Importantly, IL-6 is secreted mainly from macrophages but not T cells, and anti-IL6 receptor mAb treatment does not likely inhibit the cytotoxicity of CAR T cells [4].

## BCMA-CAR T cell therapy for multiple myeloma

Multiple myeloma (MM) is a hematological cancer derived from plasma cells. Myeloma is one of the most frequent hematological cancer. Recent advances in MM treatment are remarkable, but the cure for MM is still extremely difficult. Therefore, the development of new therapeutic drugs is needed, and CAR T cell therapy is considered promising.

Several antigens have been investigated as targets for CAR T cell therapy against MM. One promising antigen is B cell maturation antigen (BCMA). BCMA is expressed in a part of B cells, normal plasma cells, and MM cells, but not in other hematological cells including hematopoietic stem cells and other normal organs. BCMA expression is detected in most MM cases, although the expression levels of BCMA in MM cells vary from case to case. Anti-MM CAR T cell therapy targeting BCMA has been tested in several clinical trials, and some trials are now on-going. According to the results that have been recently reported from NCI’s group [5], the overall response rate was 81% (13 out of 16 patients), and very good partial response or complete response was observed in 63% (10 out of 16 patients). Median event-free survival was 31 weeks. CRS was severe in some cases but reversible. These results suggest that BCMA-CAR is very promising.

## Development of novel anti-MM CAR T cell therapy targeting activated integrin β7

**Fig. 2:**
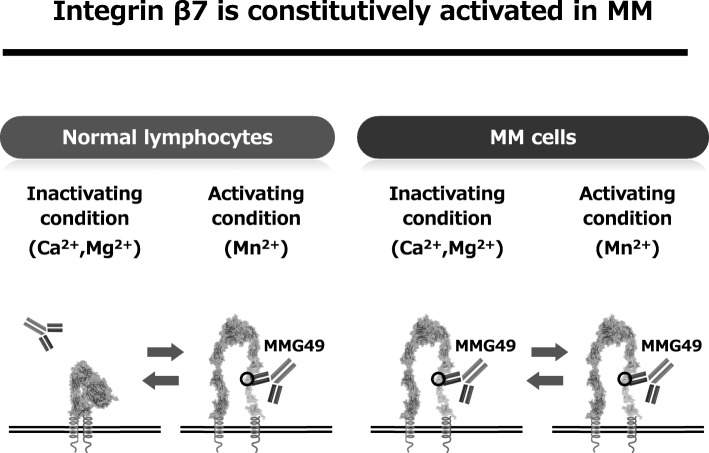
Integrin β7 is constitutively activated in MM cells and MMG49 specifically reacts with the activated conformation of integrin β7

**Fig. 3:**
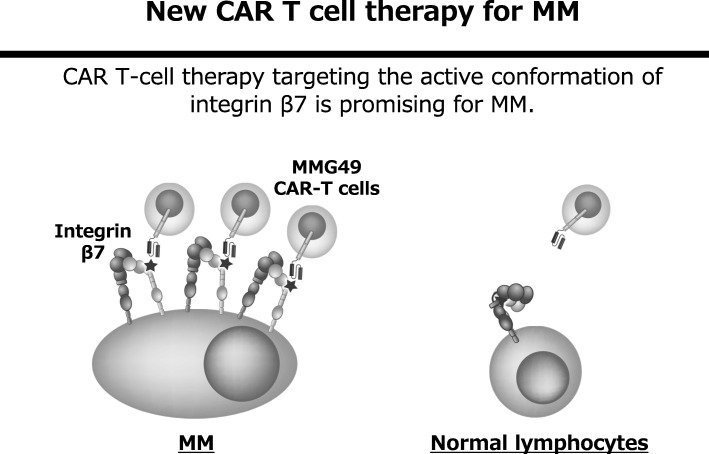
MMG49 CAR T cell provides the first clear evidence that a receptor protein with a rare but physiologically relevant conformation could serve as a target for cancer immunotherapy

We have been trying to identify MM-specific cell surface antigens. Since the search for genes and proteins specifically expressed in MM cells has already been carried out thoroughly all over the world, it seems to be extremely difficult to identify new MM-specific transcripts or proteins. However, cancer-specific antigen epitopes formed by post-translational events, such as glycosylation, complex formation, or conformational changes, might have been missed in previous screens. Indeed, a cancer-specific glyco-epitope on the Muc1 protein (Tn-Muc1) was recently shown to be an excellent target for CAR T cells against several types of cancers [6]. Such antigen epitopes could be discovered by thoroughly searching for cancer-specific mAbs and characterizing the antigens they recognize. Thus, we started developing mAbs that bind to MM cells and searching for mAbs that bind to MM cells but not to normal hematopoietic cells. As a result, an antibody called MMG49 was identified as a MM-specific antibody from more than 10,000 clones of mAbs that bind to MM cells. Next, we found that the protein to which MMG49 binds is integrin β7. Interestingly, MMG49 did not bind to normal lymphocytes although integrin β7 is certainly expressed in them. Then, we found that MMG49 binds only to the active (extended) conformation of integrin β7, but not to the inactive (bent) conformation of integrin β7. The MMG49 epitope is located in the N-terminal region of the β7 chain, which is predicted to be inaccessible in the resting integrin conformer, but exposed in the active conformation (Fig. 2). Elevated expression and constitutive activation of integrin β7 conferred high MMG49 reactivity on MM cells, whereas MMG49 binding was barely detectable in other types of cells, including normal integrin β7^+^ lymphocytes. MMG49 unlikely binds to non-hematopoietic tissues since integrin β7 mRNA is not expressed in tissues other than blood cells. Furthermore, MMG49 antigen was also highly expressed in CD19-positive clonotypic B cells, which are candidates for MM precursor cells [7], suggesting that the MMG49 antigen is a good therapeutic target for eradicating the whole MM clones. All of these results strongly suggest that the MMG49 antigen is an ideal target for CAR T cells against MM. T cells transduced with MMG49-derived CAR exerted striking anti-MM effects without damaging normal hematopoietic cells, demonstrating that MMG49 CAR T cell therapy is promising for MM. Preparation for a clinical trial is now underway. More importantly, these results provide the first clear evidence that a receptor protein with a rare but physiologically relevant conformation could serve as a target for cancer immunotherapy [8] (Fig. 3).

## Future prospects for CAR T cell therapy

CAR T cell therapy is a promising immunotherapy, but still needs to be improved. Major issues are listed below.

### Efficient trafficking of CAR T cells to the tumor sites

Efficient migration of CAR T cells to tumor sites is important for the success of CAR T cell therapy, especially for solid tumors. Some studies have shown that the introduction of chemokine receptors into CAR T cells can improve the trafficking to tumors [9, 10]. Translational research of such inventions and further basic research on the mechanism of T cell trafficking are needed.

### Avoidance of T cell exhaustion

Upon chronic antigen stimulation, T cells are exhausted [11]. Most of infused CAR T cells are likely to be exhausted after repeated stimulation by tumor cells in vivo. One way to address this problem is to use checkpoint inhibitor antibodies such as anti-PD-1, PD-L1, or CTLA-4. Another strategy is gene-editing of CAR T cells, for example, removal of the PD-1 receptor from T cells [12]. Manipulation of T cells using genome editing is becoming much easier because of the remarkable advance in genome editing technology [13]. However, it has recently been shown that fully exhausted T cells cannot be reversed by PD-1 blockade alone [14, 15]. Thus, the additional genetic/epigenetic modification should be a need for avoiding exhaustion of CAR T cells.

### Control of T lymphocyte metabolism

It has been clarified that metabolism is important for T cell function. Tumors can inhibit antitumor immunity by affecting T cell metabolism by nutrient depletion [16]. In the tumor microenvironment, tumor cells compete with T cells in the usage of glucose [17]. In addition, due to potassium released from dead tumor cells, the level of potassium ion in the tumor microenvironment is 5- to 10-fold higher than that in the bloodstream, which strongly inhibits T cell activation [18]. These findings suggest that it is possible to perform more effective CAR T cell therapy by controlling T cell function via control of cell metabolism.

### Persistence of CAR T cells

Persistence of CAR T cells predicts durable clinical effects in patients with hematologic malignancies [2]. In clinical trials with CAR T cells that lack clinical efficacy, poor T cell persistence was reported [19]. The molecular design of CARs is likely to strongly influence T cell expansion and persistence [20]. The signals delivered by the current CAR constructs may not be optimal for long persistence of CAR T cells, and many researchers are trying to develop novel CAR constructs that can induce long persistence of CAR T cells. CAR constructs currently being tested in the clinic contain a CD3z (TCR signaling) domain and co-stimulatory domain(s). Kagoya et al. recently reported a novel CAR construct that encodes a truncated cytoplasmic domain from the interleukin (IL)-2 receptor beta-chain (IL-2Rbeta) and a STAT3-binding tyrosine-X-X-glutamine (YXXQ) motif, together with CD3z and CD28 domains [21]. This novel CAR can transduce antigen-dependent cytokine signaling in addition to TCR engagement and co-stimulatory signals, which promoted their proliferation and prevented terminal differentiation.

### Development of off the shelf CAR T cells

The extremely high cost of CAR T cell therapy is a critical issue. One major reason for the high cost is that CAR T cells have to be made for each patient. To address this issue, many researchers are now trying to develop “off the shelf” CAR T cells, which are established from a donor and can be used for many patients. One strategy is allogenic donor-derived CAR T cells. T cell receptor, which may cause an allogenic immune reaction, can be deleted using genome editing techniques [22]. Another promising strategy is iPS cell-derived CAR T cells. Some researchers have already succeeded in producing functional T cells from iPS cells [23, 24] and are now trying to use them for the source of CAR T cells.

## Conclusion

CAR T cells are a very promising treatment for cancers. Because of its potential for eradicating the targets, CAR T cells are expected to be a good tool for curing cancer patients. After the big success of CD19-CAR T cells, many researchers are working on CAR T cells against MM. BCMA-CAR is an excellent candidate. We also developed an anti-MM CAR T cells that can target not only MM plasma cells but also immature clonotypic B cells and will test it in a clinical trial very soon.
